# Biogeographical Distribution of Bacteria in Soils with Identical Agricultural Practices: Impacts of Environmental Factors

**DOI:** 10.1007/s00284-025-04404-w

**Published:** 2025-08-03

**Authors:** Muiz O. Akinyemi, Sinawo Tsipinana, Kazeem A. Alayande, Maphala Mokubedi, Rasheed A. Adeleke

**Affiliations:** 1 Unit for Environmental Sciences and Management, North-West University, Potchefstroom, 2520 South Africa; 2 Leeds Institute of Health Sciences, University of Leeds, Leeds, UK

## Abstract

Soil microbial communities are key drivers of plant health and productivity, influencing nutrient cycling and energy flow. This study investigated the impact of environmental variables on the bacterial communities within the rhizospheres of maize and soybean, two economically important crops, across contrasting South African climates with identical agricultural practices. Using 16S rRNA amplicon sequencing, we conducted a comparative analysis of rhizosphere bacterial communities in the semi-arid steppe of Free State and the subtropical region of Mpumalanga. In the semi-arid steppe, *Actinobacteriota* (36.5%), *Pseudomonadota* (24.4%), and *Chloroflexi* (12.8%) were dominant, while the subtropical region had higher proportions of *Actinobacteriota* (34.8%), *Pseudomonadota* (27.6%), and *Acidobacteriota* (12.6%). Our findings indicate that while a core bacterial community persists across regions, environmental factors significantly influence the diversity of non-core taxa. For instance, in Mpumalanga, correlation analysis suggested that the abundance of families like *Burkholderiaceae* and *Symbiobacteraceae* were influenced by moisture and minimum daily temperature while in Free State, families such as *WS2* and *Legionellaceae* were influenced by maximum daily temperature, soil pH, and texture. These findings underscore the influence of climate and soil properties on the rhizosphere microbiome and highlight the importance of understanding these patterns for enhancing crop production in changing environmental conditions.

**Supplementary Information:**

The online version contains supplementary material available at 10.1007/s00284-025-04404-w.

## Introduction

The composition of a plant microbial community plays a critical role in its ability to thrive, particularly under adverse circumstances. In semi-arid regions such as South Africa, the efficiency of plant acquisition of nutrients is diminished due to drought conditions, making the survival of plants heavily reliant on their adaptive strategies. One such strategy involves the dependence on rhizosphere microorganisms. The rhizosphere, which is the soil area immediately surrounding plant roots, is home to a dynamic microbial community. These microbes interact with the plant, and some contribute to the plant’s resilience against factors that impede growth. Beneficial microbes within the rhizosphere are essential for carbon capture, ecosystem health maintenance, disease-causing organisms’ management, and nutrients recycling in land-based ecosystems. Therefore, it is crucial to understand the structure, environmental interactions, movements, and functions of these rhizosphere microbial groups. This insight is key to effectively employing rhizosphere microorganisms of the development of sustainable agricultural practices.

Plants can modify soil properties to adapt and ensure their survival under adverse conditions. As plants progresses through their growth and development, various allelochemicals, including photosynthates, secondary metabolites such as antimicrobial compounds, and defensive phytohormones [1], are released from the roots through exudation, secretion, and deposition, enriching the rhizosphere with nutrients compared with the surrounding bulk soil. Microorganisms can use these compounds as substrates, resulting in increased microbial biomass and activity around the roots. The establishment of a dense microbial community in the rhizosphere, driven by nutrients secreted by plant roots, is known as the rhizosphere or plant effect. Similar to microbial communities associated with other parts of plant, microbes within the rhizosphere are subject to various environmental factors such as climate, soil salinity, and soil ph. For example, previous report has shown that soil texture [2], pH [3] precipitation [4], temperature [5], humidity [6], rainfall [7], and nutrient content [8] play an important role in the orientation and modulation of soil microbial communities and the natural ecosystems [6]. Hence, the effects of environmental factors as a global change factor can cause microbial species range expansions and contractions either directly or indirectly [9]. While climate change may not necessarily result in a decrease in soil microbe diversity, it may have a greater extinction effect on uncommon microbial species than on common ones [10]. Some of these species with lower abundance even serve important functions in the microbial ecosystem as a whole [11]. All of these have local- and, potentially, global-scale implications on soil health indices, vegetation distribution and soil microbiomes, thereby threatening food security [12].

This study set out to investigate the effects of soil and climatic conditions on the richness and composition of bacterial communities in the rhizosphere of two commercially significant crop species: maize and soybean. The study was carried out in two South African provinces, Free State and Mpumalanga, known for their high agricultural productivity of selected crops. This study builds upon an earlier investigation conducted in the Free State province, which examined the effects of fertilizer use, as well as tillage and no-till farming practices, on microbial populations in the rhizosphere of soil in a maize-soybean crop rotation system. We refrained from addressing these aspects in the results provided here; nevertheless, we incorporate a sampling method to ensure that the study could be replicated. The specific aim of this study was to investigate the impact of environmental variables, particularly climate and soil properties, on the assembly, structure, and diversity of bacterial communities within the rhizospheres of maize and soybean across contrasting South African climates.

## Materials and Methods

### Sampling Site

South Africa (22°−35°S) encompasses two key maize/soybean production regions. Maluti-a-Phofung (Free State: 28° 25′ S, 28° 56′ E) borders Lesotho on a plateau (~ 1000 m). This cold, semi-arid steppe has dry winters, dark clay soils, ~ 800 mm annual rainfall, and ~ 17 °C. As South Africa's'breadbasket', Free State uses 2.5 million hectares for crops. Gert Sibande (Mpumalanga: 26° 45′ S, 30° 13′ E) is the province's largest district. Its eastern highveld (~ 1600 m) is subtropical highland mesic grassland with dry winters, > 700 mm rainfall, < 24 °C, and sandy soils (< 15% clay). Mpumalanga dedicates 943,163 hectares to crops. Agriculture is a key economic sector in both regions, justifying their selection for study.

### Field Plots

A detailed description of the experimental set up of our larger study, in which the data herein were obtained, were previously published [13]. Briefly, an illustrative diagram of the randomized complete block design with a 2 × 2 × 3 strip split plot treatment structure replicated three times. The main plots were made up of two tillage systems (conventional tillage (CT) and No-till (NT)), whilst the two crops soybean and maize made up the sub-plots and three phosphorous fertilizer application rates (0, 30 and 60 kg ha^–1^) made up the sub-sub plots. Each plot consisted of six 7 m long soybean rows with an inter and intra-row spacing of 60 and 5 cm, respectively (gross plots), targeting a population of 300,000 seeds per hectare. The net plots consisted of four middle rows of the gross plots. Similar dimensions were used for the maize plots at a spacing of 60 cm × 30 cm. The study started on an untouched land in December 2014. In 2015 growing season, there was no experiment conducted due to drought and the trial commenced again in 2016.

### Weather Data Acquisition

Climatic data for both locations were sourced from land-based stations operated by the South African Weather Service (https://www.weathersa.co.za/). The station in the Free State was situated in Kroonstad, while the station in Mpumalanga was in Mbombela. Supplementary data 1 contains the climate data for the entire duration of the experimental growth periods in 2014 and 2016, including daily averages.

### Rhizosphere Soil Sampling

A total of thirty-six composite soil samples were collected from two study regions, each containing six plots, resulting in twelve plots total. Rhizosphere soil was systematically sampled at crop maturity, one day before harvest. Within each plot, three samples were taken near maize and soybean plants (for a total of three samples per plot), all from the top 0–15 cm of soil. Importantly, each of these individual samples was created by combining three subsamples collected together to form one composite sample. Approximately 1 kg of soil was collected for physicochemical analysis using a sterile soil auger, while 50 g of soil was gathered for microbiome analysis using a sterile spatula. Each replicate was derived from three distinct points within the plot, targeting the plot's opposite ends and central area. These spatially distributed samples were then combined to create a composite replicate sample. This approach minimized localized sampling bias and provided a comprehensive representation of the plot’s soil microbiome and physicochemical characteristics. Samples were transported to the laboratory in insulated boxes with ice packs, stored at −20 °C upon arrival, and processed within seven days. Before analysis, plant debris and roots larger than 2 mm in diameter were aseptically removed. While this composite sampling approach minimizes localized bias and provide a plot-level representation of rhizosphere properties, it may underestimate micro-spatial heterogeneity arising from root-soil interactions or localized biogeochemical processes. Pooling subsamples could further dilute rare microbial taxa or transient physicochemical gradients; however, this trade-off was necessary to prioritize broad comparability across plots and regions.

### Analyses of Soil Physicochemical Parameters

Standard chemical procedures previously described by Adeleke et al. [13] were used to determine the physicochemical parameters of soil. Briefly, total nitrogen (N) was determined using the Kjeldahl technique. Soil sample were digested with sulfuric acid (H₂SO₄), potassium sulfate, and selenium. The mixture was then heated at 370 °C until colourless. The resulting ammonium was distilled with sodium hydroxide-thiosulfate and collected in boric acid solution, which was then titrated with standardized sulfuric acid. The total nitrogen (TN) was calculated using the following formula:

$${\text{TN}}\;\left( {\text{mg/L}} \right) = \left[ {\left( {A - B} \right) \times N \times 14 \times 1000} \right] \div S,$$

where *A* = mL of standard H₂SO₄ used for sample titration, *B* = mL of standard H₂SO₄ used for blank titration, *N* = Normality of H₂SO₄, 14 = Atomic weight of nitrogen, *S* = mL of original sample.

Total phosphorus was measured using the Murphy-Riley colorimetric method. The soil sample was first digested, and then the phosphorus content was determined by measuring the absorbance of the molybdenum blue complex at 880 nm using a spectrophotometer.

Soil pH was measured using a Metrohn pH meter (692, Herisau, Switzerland). A 1:10 soil to deionized water mixture was prepared, stirred, and allowed to settle before the pH reading was taken.

Total potassium concentration was determined using a flame atomic absorption spectrometer. The soil sample was first extracted with 1 M ammonium acetate, and then the extract was analysed using the spectrometer.

Particle size distribution was measured using the hydrometer technique. The soil samples were ground, sieved (pore size:2 mm) and treated with hydrogen peroxide to oxidize organic matter that can interfere with particle dispersion. Thereafter, sodium hexametaphosphate solution was added to the treated soil. The prepared sample was transferred to a 1000 mL sedimentation cylinder and thoroughly mixed with distilled water using. Hydrometer readings were taken at specific time intervals, measuring the density of the soil suspension. Temperature and density readings of the suspension and reagent blanks were taken after 40 s and 2 h. These measurements were used to calculate the proportions of sand, silt, and clay using the following formula:

$$\% {\text{ clay}} = {\text{hydrometer}}\;{\text{reading}}\;{\text{at}}\;2\; {\text{h}} - {\text{blank}}\;{\text{reading}}\;{\text{at}}\;2\; {\text{h}} \times 100 \div {\text{weight}}\;{\text{of}}\;{\text{soil}}\;{\text{sample}}$$

$$\% {\text{ silt}} = {\text{hydrometer}}\;{\text{reading}}\;{\text{at}}\;40\;{\text{s}} - {\text{blank}}\;{\text{reading}}\;{\text{at}}\;40\;{\text{s}} \times 100 \div {\text{weight}}\;{\text{of}}\;{\text{soil}}\;{\text{sample}} - \% {\text{clay}}$$

$$\% {\text{sand}} = 100\% - \% \left( {{\text{clay}} + {\text{silt}}} \right)$$

### DNA Extraction, Sequencing, and Microbiome Analyses

Soil DNA from the 36 samples were extracted and purified using DNeasy PowerSoil Kit (Qiagen, Hilden, Germany). DNA purity and concentrations were estimated by fluorometric quantification (Qubit 2.0, Invitrogen, CA, USA). Bacterial 16S rRNA genes were amplified using Illumina-MiSeq barcoded forward (341 F) and reverse (805 R) primer pairs. Briefly, amplicon PCR was performed using 2.5 μL of microbial DNA, 5 μL each of 1 µM forward and reverse primers, and 12.5 μL of 2 × KAPAHiFiHotStart ReadyMix in a 25 μL total reaction volume. Amplification was carried out in a thermal cycler with an initial denaturation at 95 °C for 3 min, followed by 25 cycles of denaturation at 95 °C for 30 s, annealing at 55 °C for 30 s, extension at 72 °C for 30 s, a final extension at 72 °C for 5 min, and a hold at 4 °C. The PCR amplicons were purified with AMPure XP magnetic beads (Beckman Coulter, CA, USA), indexed using Nextera XT primers (Illumina Inc.) repurified AMPure XP magnetic beads. The purified library was quantified, normalized, pooled in equimolar concentrations, and denatured in 0.2 N NaOH. Paired end (2 × 300 bp). The partial 16S rRNA gene libraries were prepared and sequenced at the Agricultural Research Council in Pretoria, South Africa. The process utilized the Nextera v3 kit, following the manufacturer's guidelines, and employed the Illumina-MiSeq platform (Illumina Inc., San Diego, CA, USA) for sequencing. Sequence data generated in this work is available at the NCBI Sequence Read Archive under Bio Project number PRJNA667129. The quality of sequence reads was determined using FastQC v0.12 (Andrews 2010). Poor-quality sequences (i.e., 200 bp sequences with an average quality score of less than 25 and ambiguous characters) and adapter sequences were removed using in Trimmomatic 0.40 [14]. The resulting high quality small subunit rRNA gene data were processed and analysed as previously described by Akinyemi et al. [15], using the QIIME software package [16]. The sequence reads were grouped into amplicon variants (ASVs) using DADA2 after marginal sequences and sequencing artefacts were removed [17]. Taxonomic classification of the representative sequences was based on 97% sequence similarity using the Scikit-learn feature classifier with the 0.7% default confidence threshold against the SILVA v132 reference database for bacterial V3–V4 region, and taxa with relative abundance greater than 0.01% were considered present. Thereafter, sequences classified as archea, fungal, chloroplasts or mitochondria were removed using the SILVA reference database (v138.1) within QIIME2 (q2-feature-classifier). The abundance-based richness (i.e., Chao1 and ACE) and diversity (i.e., Fisher, Shannon, Simpson) indices also known as alpha-diversity indices were analysed and visualized using the ‘phyloseq’ package v1.16.2. Using the ‘agricolae’ package v1.3–7, statistical comparisons between the alpha-diversity indices was determined by the non-parametric Wilcoxon signed-rank test with adjusted *P* values using the Benjamini–Hochberg method. To determine the similarities in microbial communities between the sampling sites, beta diversity analysis was performed using the Analysis of Group Similarities statistics (ANOSIM) and Bray–Curtis distance method available in the ‘vegan’ package v2.6–4. The beta diversity outputs were visualized as Non-Metric Multidimensional Scaling (NMDS) ordinations and the unweighted pair-group method with arithmetic means (UPGMA) hierarchical clustering. Identification of bacterial species characterizing differences between soil sample groups was determined using Linear discriminant analysis effect size (LEfSe) at alpha level set at 0.05, clade label level set at 5 for the top 50 dominant taxonomic groups. Association between environmental parameters (e.g., soil and weather parameters) and bacterial communities was visualised with ordinates on a redundancy analysis (RDA) plot using ‘vegan’ package v2.6–4.

### Statistical Analysis

R statistical software version 4.2.1 was used for statistical analyses. Levene's test was used to assess homogeneity of variances across microbial community groups in both sample sites, which is crucial for subsequent analytical approaches. A non-parametric Kruskal–Wallis analysis of variance (ANOVA) was applied to assess differences in bacterial phyla relative abundance. The Mann–Whitney *U* test was used to statistically examine differences between environmental parameters from both sample sites. Multiple regression was used to determine associations between environmental factors and microbial populations. All statistical tests were evaluated at a significance level of *P* < 0.05, with differences between means and correlations considered statistically significant.

## Results

### Sequencing Summary, Bacterial Richness, and Diversity

A total of 5,048,294 paired end reads, with an average of 144,237 and range between 37,569 and 270,614 sequences were generated (Supplementary Fig. S1). After quality filtering, denoising, merging, chimera screening and deletion of chloroplast and mitochondrial DNA, an average of 95,319 reads per sample were obtained (Supplementary Fig. S1). The filtered reads were grouped into 601 amplicon sequence variants. Despite a large fraction of amplicons assigned to plastid 16S rDNA, rarefaction analysis showed sequencing depth was high enough to represent the observed ASV (Supplementary Fig. S2).

**Fig. 1:**
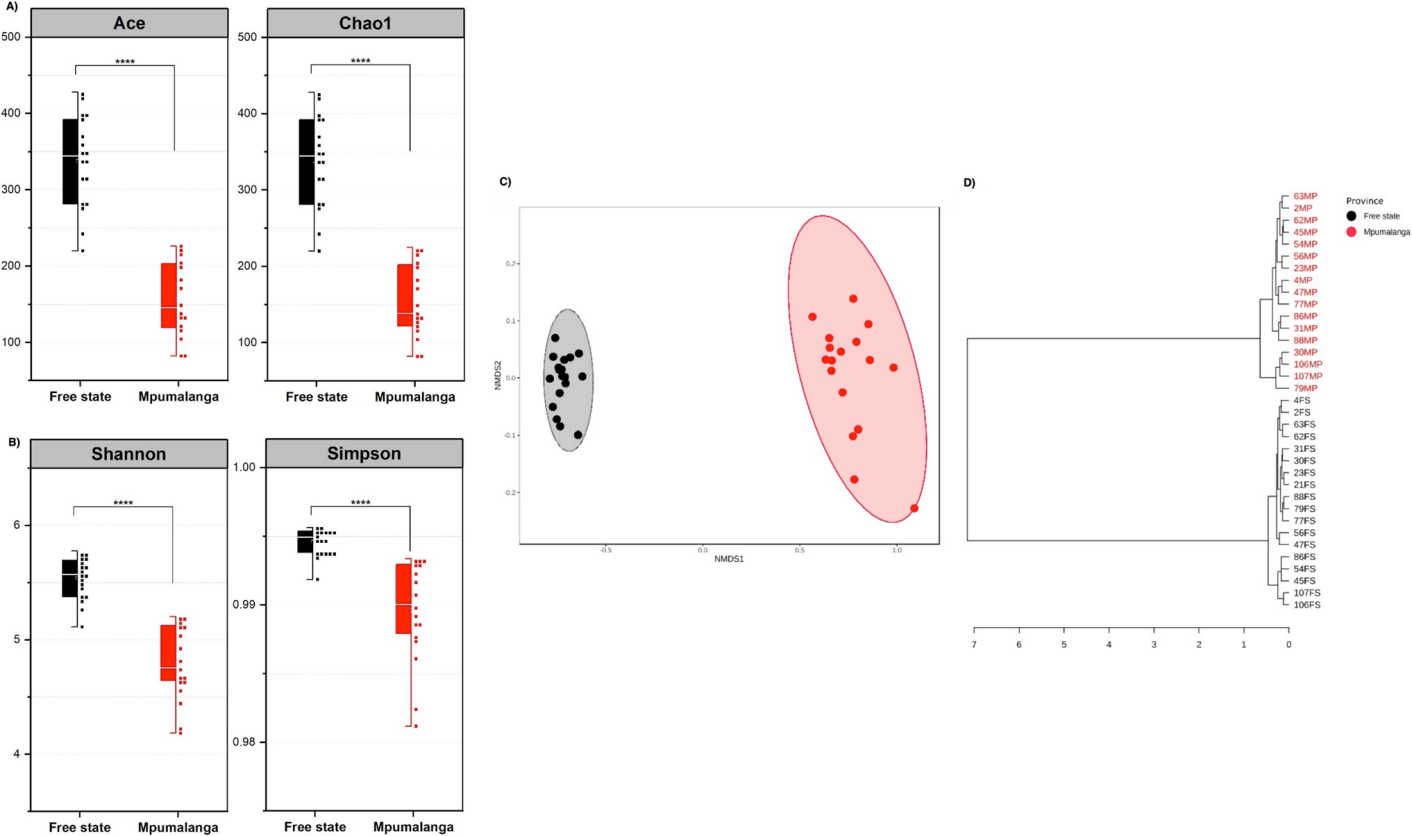
Microbial diversity indices representing **a** Chao1 and ACE richness estimator and **b** Simpson and Shannon diversity estimators indexed for rhizosphere soil from Free state and Mpumalanga province. **c** Non-metric Multidimensional Scaling (NMDS) and **d** cluster dendrograms of microbial communities in soil from Free state and Mpumalanga province based on Jaccard Index

The diversity indices of two provinces were compared using estimators of richness and diversity, as depicted in Fig. 1a, b. The semi-arid steppe location (SAS-FS) in the Free State province exhibited a notably higher species richness than the subtropical highland (SH-M) in Mpumalanga, according to ACE (*P* < 0.001) and Chao1 (*P* < 0.001) indices. Furthermore, there was a significant variance in microbial diversity and evenness between the sites, as indicated by Shannon’s (*P* < 0.002) and Simpson’s (*P* < 0.003) indices.

The analysis of beta diversity revealed distinct variations in the bacterial populations within the rhizospheres of the two areas, as determined by the Jaccard index. These variations were depicted through non-metric multidimensional scaling (NMDS) as shown in Fig. 1c. The adonis/ANOSIM test confirmed that the differences in soil samples from the two regions were statistically significant, with a *P* value less than 0.001. Additionally, the dissimilarities between the two microbial communities were further supported by the UPGMA cluster dendrograms illustrated in Fig. 1d.

### Microbiome Composition and Differentially Represented Taxa

**Fig. 2:**
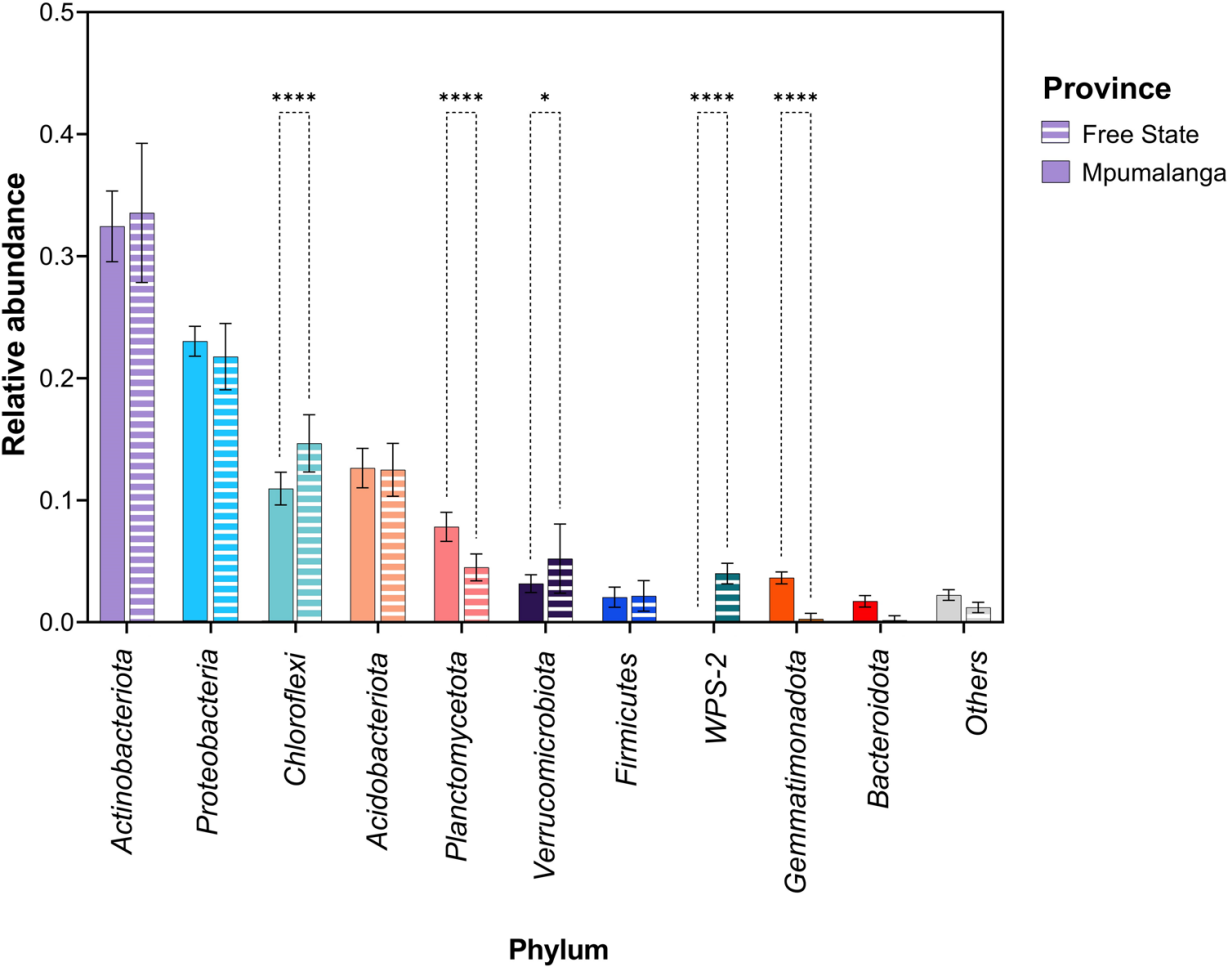
Bar plot visualization of relative abundance of taxa at the phylum level (> 1% relative abundance) in soil sample from Free state and Mpumalanga province, South Africa

Bacteria sequences found in this study with relative abundance greater than 1% were categorized into 10 phyla (Fig. 2). *Actinobacteriota* emerged as the most abundant group, accounting for 32.9% of the 16S rRNA gene sequences. Following was *Pseudomonadota* at 22.5%, *Chloroflexi* at 12.9%, *Acidobacteriota* at 12.5% and *Planctomycetota* at 6.1%. Overall, these five phyla were the most prevalent. All phyla with a relative abundance greater than 1% were observed in SAS-FS, with *Actinobacteriota* (36.5%), *Pseudomonadota* (24.4%) and *Chloroflexi* (12.8%) as the predominant groups. Conversely, the phylum WPS-2 was absent in SH-M, where *Actinobacteriota* (34.8%), *Pseudomonadota* (27.6%) and *Acidobacteriota* (12.6%) were most prevalent. The variance analysis of the phyla detected in both provinces revealed that *Planctomycetota* (*P* < 0.001) and *Gemmatimonadota* (*P* < 0.001) were significantly more abundant in the subtropical highland region, whereas *Chloroflexi* (*P* < 0.001), *Verrucomicrobiota* (*P* < 0.05), and *WPS-2* (*P* < 0.001) were notably more abundant in the semi-arid steppe region, as illustrated in Fig. 2.

**Fig. 3:**
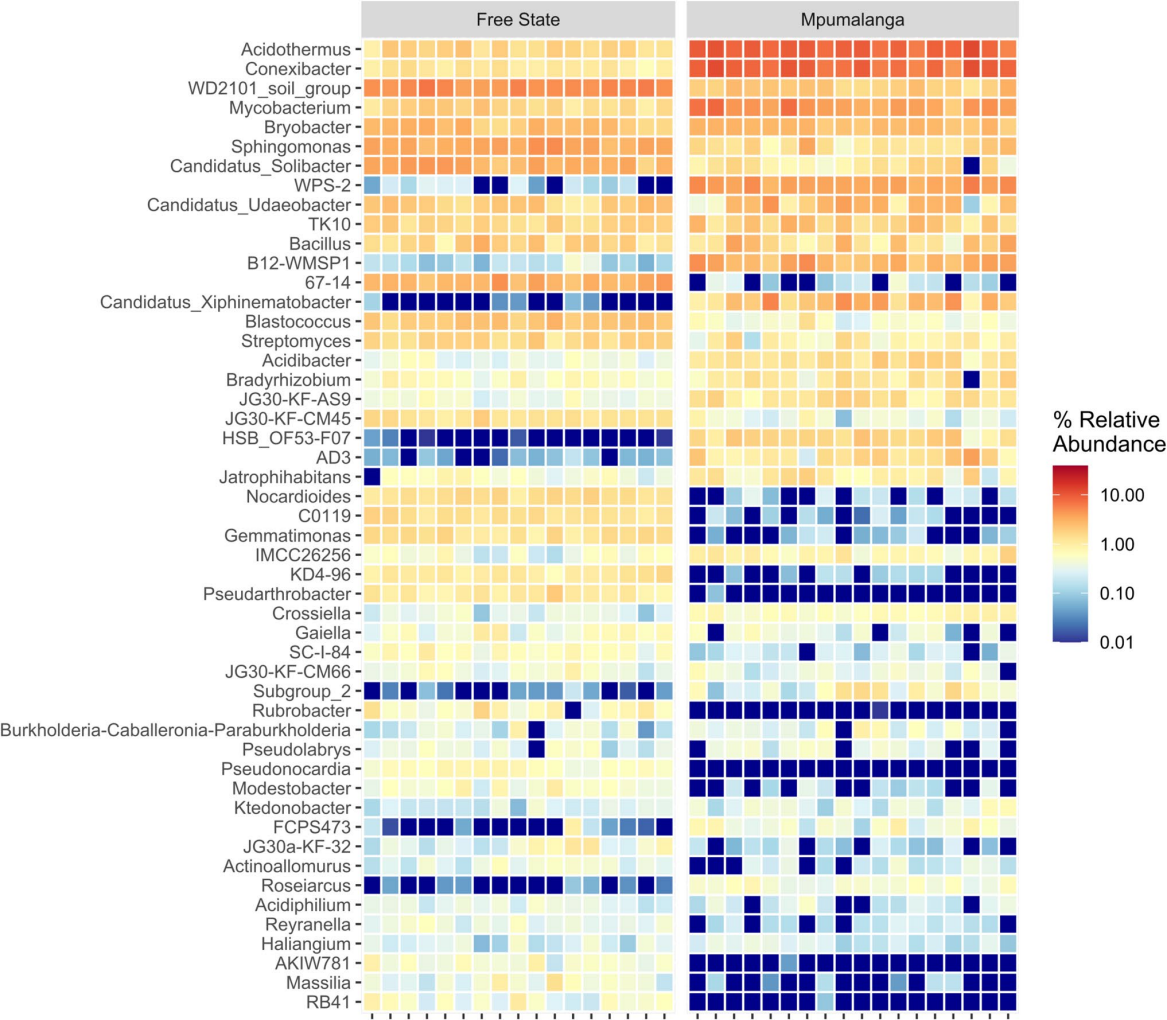
Heat map visualization of taxonomic composition at the genus level revealing the selected classification of top 50 dominant bacteria in soil sample from Free state and Mpumalanga province, South Africa

Across all rhizosphere soil samples, a diversity spanning 31 classes, 67 orders, 90 families, and 113 genera were identified, however, only the top 50 abundant genera are illustrated in Fig. 3. Specifically, in SAS-FS, 100 taxonomically classified genera were discovered. The ten most abundant genera, in order of their relative abundance, included: *Sphingomonas, WD2101 soil group, Blastococcus, Candidatus_Solibacter, Bryobacter, Streptomyces, Acidothermus, 67-14, Candidatus Udaeobacter* and *Bacillus*, as shown in Fig. 3.

In contrast, 66 genera with taxonomic designations were detected in SH-M, with the ten most prevalent being *Conexibacter, Acidothermus, Mycobacterium, WPS-2, Candidatus Xiphinematobacter, Bryobacter, Candidatus Udaeobacter, Sphingomonas, Bacillus* and *B12 WMSP1*, also detailed in Fig. 3. It is noteworthy that approximately 24.5% of the genera found in the study sites were classified as uncultured bacteria while 7.5% of the ASVs where unassigned to a taxon.

**Fig. 4:**
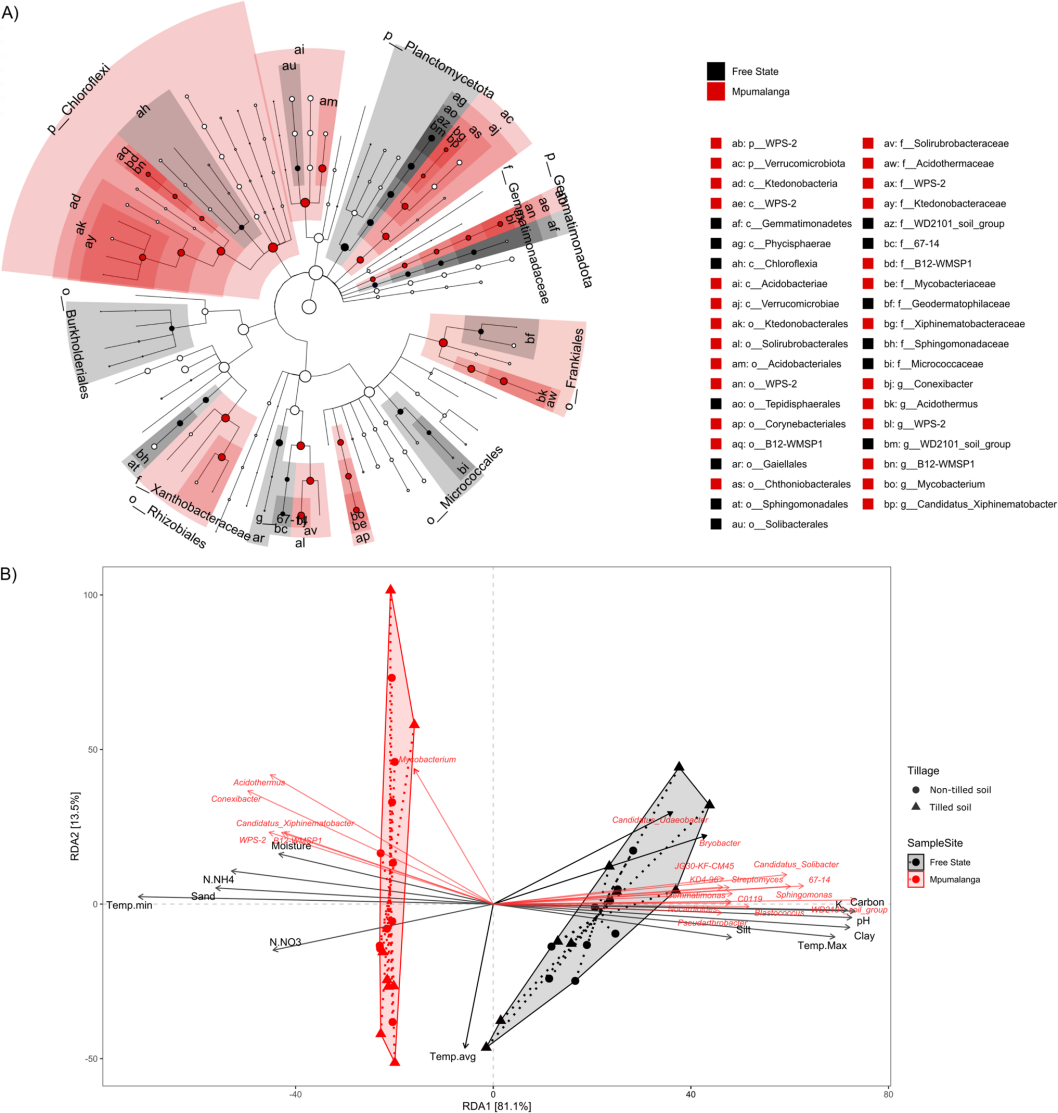
**a** Linear discriminant analysis effect size (LEfSe) of the bacterial communities with an LDA score higher than 4.0 and *P* values less than 0.05. Cladograms indicate the phylogenetic distribution of microbial lineages associated with each province. Circles represent phylogenetic levels from kingdom to genus. Abbreviations: p, Phylum; c, Class; o, Order; f, Family; g, Genus; s, Species. **b** Redundancy analysis showing the relationship between environmental variables and bacteria communities in Free state and Mpumalanga sampling sites

Linear discriminant analysis (LDA) effect size (LEfSe) analysis was performed to identify distinguishing microbes in the rhizosphere communities between both regions, using biomarkers with LDA scores exceeding 4, as shown in Fig. 4a. These biomarkers demonstrated notable differences in the relative abundance of key genera. Genera such as *Acidothermus, B12-WMSP1, Candidatus Xiphinematobacter, Conexibacter, Mycobacterium* and *WPS*−2 were found to be more prevalent in the subtropical highland area. On the other hand, genera including *67-14, Blastococcus, Candidatus Solibacter*, and *WD2101 soil group* were predominantly found in the semi-arid steppe region.

### Bacterial Community Structure as a Function of Tillage Practice, Fertilizer Management and Environmental Factors

A comprehensive analysis of how tillage and fertilizer management impact the microbial profiles in samples from SAS-FS has been documented in a previous study. In summary, the practice of tillage seemed to play a role in shaping the microbial community structure within the semi-arid steppe region. In the non-tilled plots, the biomarkers identified were *Pseudomonadota, Actinobacteria, Planctomycetes, Verrucomicrobia, Cyanobacteria* and *Acidobacteria*. Conversely, in the tilled plots, the biomarkers were *Nitrospirae, Chloroflexi, Firmicutes*, and *Bacteroidetes*. Furthermore, at the genus level, *Lysobacter, Dyella, Amycolatopsis, Sphingomonas, Haliangium, Dactylosporangium* and *Pirellula* stood out as biomarkers in the non-tillage treatment. Random forest-based test for biomarker detection in samples from SH-M indicated that in non-tilled plots, the biomarkers included *Patescibacteria, Cyanobacteria, Abditibacteriota, GAL15, Myxococcota*, and *WPS2* at the phylum level and *Paraburkholderia, Acidibacter, Flavisolibacter, Bradyrhizobium, Thermosporothrix* and *Dyella* and the genus level (Fig. 4b). In contrast, tilled plots had the phyla *Planctomycetota, Firmicutes, Bacteroidota, Latescibacterota, Chloroflexi* and *RCP2_54* and genus *SC_I_84, Subgroup 10KF_JG30_B3, Chujaibacter, Edaphobacter, Modestobacter* and *Subgroup 2* as biomarkers (Fig. 4b).

Our earlier findings from SAS-FS demonstrated that applying phosphorus fertilizer significantly impacted bacterial diversity. However, this significant effect was not observed in the SH-M samples. To understand the reasons behind these differing outcomes, environmental factors (weather and soil physiochemical properties) were considered and further investigated as the potential underlying cause.

**Fig. 5:**
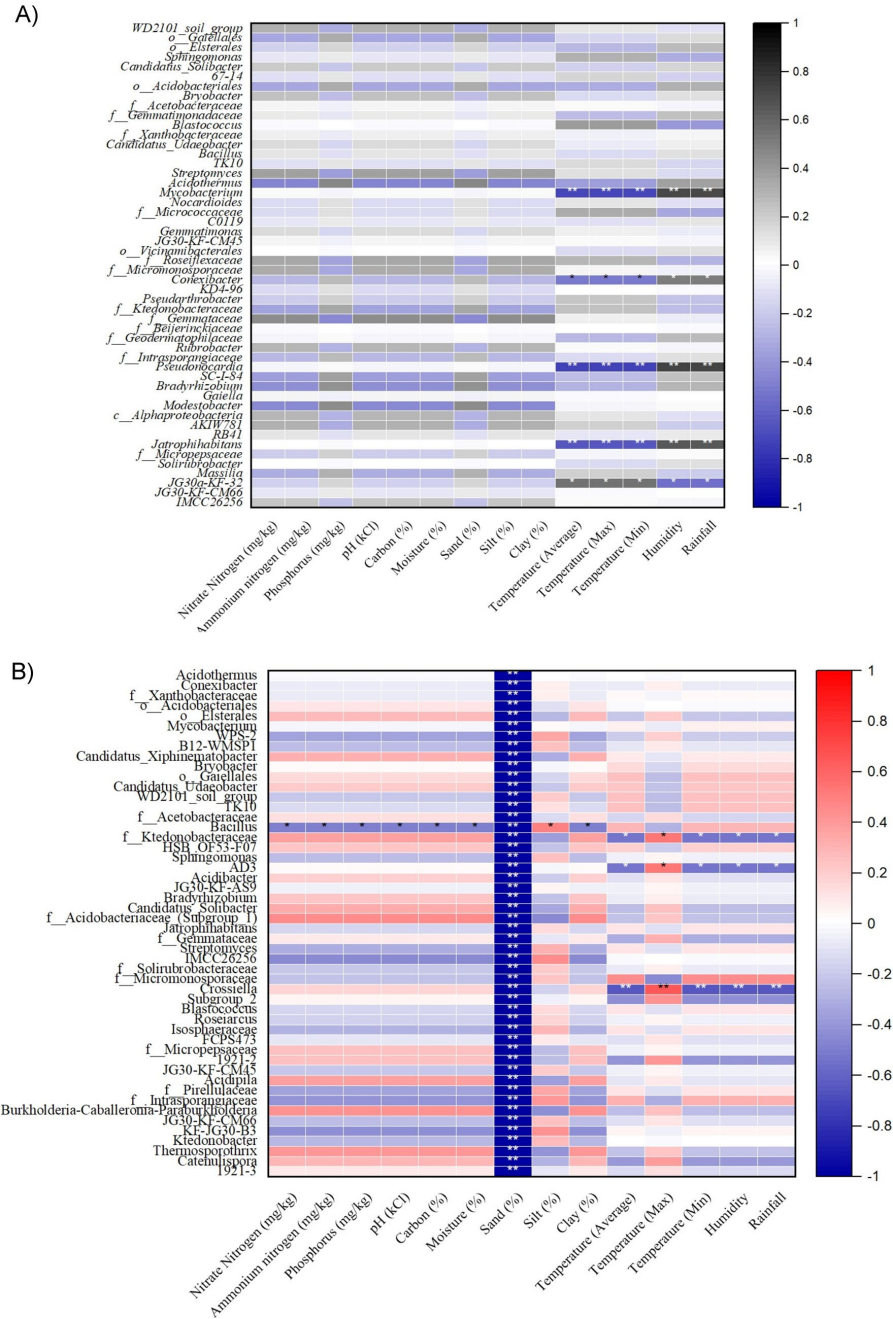
Heat map showing correlation between top abundant bacterial genera in soil with soil and climatic factors from Free State (**a**) and Mpumalanga (**b**) province. Colours indicate the *r* values of Spearman’s rank correlation coefficients. *= *P* ≤ 0.05, **= *P* ≤ 0.0

In the planting seasons of 2014/15 and 2016/17, notable variations were observed in weather parameter trends between SAS-FS and SH-M regions. We observed significant differences in daily average values for maximum, minimum and average humidity, as well as maximum and minimum temperatures, wind speed and average dew point from planting to harvest (*t* test, *P* < 0.05). During the four weeks preceding harvest, the minimum temperatures, dew point, and both maximum and minimum humidity were notably lower in the SH-M region compared to SAS-FS (*t* test, *P* < 0.05) with mean values of 17.1 °C, 11.4 °C, 84.1, 42.6 and 11.5 °C, 2.4 °C, 82.3%, 25.4%, respectively. Consequently, specific weather factors exhibited differences between the provinces in the final third of the planting period (approximately one month) in our study. Notably, mean daily precipitation was similarly low (< 0.1) or absent in both provinces (Supplementary data 1), with severe drought conditions during the 2015/16 season. To test environmental factors’ effect on the microbial communities, RDA was performed and visualized as a biplot shown in Fig. 5. Only those factors with significant values (*P* ≤ 0.05) were plotted. The cumulative interpretation rates of the first two-ranking axis of the RDA explained 94.6% of the total variance between both bacterial communities. Result shows that in SH-M, the moisture level and minimum temperature, in conjunction with sand content in the soil, had significant impact on the variability of rhizosphere bacteria composition. Conversely, in SAS-FS, the soil pH, clay, and silt content, along with the maximum temperature, played a crucial role in shaping the microbial community. Therefore, we sought to determine which families and differentially abundant genera significantly correlated with these variables. The relationship between rhizosphere bacteria at harvest and the daily average environmental variables during the four weeks leading up to harvest is presented in Supplementary Data 2. Only statistically significant (*P* < 0.05) correlations are reported here.

In the SH-M region, the families *Ktedonobacteraceae*, *RCP2-54*, *Alicyclobacillaceae*, *Burkholderiaceae*, *Rhizobiaceae*, *Koribacteraceae*, and *Acidimicrobiaceae* showed negative associations with sand content and minimum temperature, respectively. Conversely, *Ktedonobacteraceae*, *Burkholderiaceae*, *RCP2-54*, *Symbiobacteraceae*, and *IMCC26256* were positively correlated with moisture and minimum temperature, respectively. Additionally, *Bacillaceae*, *KF-JG30-B3*, and *Kapabacteriales* exhibited negative correlations with moisture content but positive correlations with sand content. In the SAS-FS region, several bacterial families displayed negative correlations with specific environmental factors. For instance, approximately 25 families, including *Xiphinematobacteraceae*, *Gemmataceae*, *Chloroflexaceae*, *Enterobacteriaceae*, and *Candidatus_Uhrbacteria*, were negatively associated with pH. *Microbacteriaceae* showed a negative correlation with clay content, while *Gemmataceae* and *Obscuribacteraceae* were negatively correlated with maximum temperature. On the other hand, positive correlations were observed in certain families: *Kineosporiaceae* and *Streptosporangiaceae* with soil pH; *Diplorickettsiaceae*, *mle1-27*, *Anaerolineaceae*, and *Rhodobacteraceae* with silt content; *Devosiaceae*, *Hyphomicrobiaceae*, *mle1-27*, *Saccharimonadaceae*, *Rhodobacteraceae*, and *WS2* with clay content; and *Solirubrobacteraceae*, *Intrasporangiaceae*, *Legionellaceae*, *Elsteraceae*, *Kapabacteriales*, *Sporomusaceae*, and *OLB14* with maximum temperature. Most of the genera with higher abundance in SH-M exhibit inverse relationships with nearly all environmental variables in SAS-FS. Noteably, *B12-WMSP1, Conexibacter* and *Mycobacterium* showed significant (*P* < 0.05) negative association with sand content. In contrast, no significant (*P* > 0.05) association was observed between the dominant genera in SH-M and its environmental variables (Supplementary Data 2).

## Discussion

Soil harbors diverse microbial populations shaped by biotic and abiotic processes. To understand environmental influences on the vital South African cash crops maize and soybean, we characterized their rhizosphere microbiomes immediately post-harvest from maiden farmlands during the 2014/15 and 2016/17 seasons in Free State and Mpumalanga provinces. Despite notable differences in overall rhizosphere bacterial communities between ecosystems, they shared identical core microbial phyla: *Actinobacteriota, Acidobacteriota, Chloroflexi, and Pseudomonadota*. These core phyla are consistently associated with legume rhizospheres globally [18, 19] and specifically in South African provinces [20–23], suggesting a key role in supporting leguminous plant growth or maintaining rhizosphere structure. Furthermore, genera within these phyla such as *Rhodococcus, Streptomyces, Bryobacter, Chloroflexus, Bradyrhizobium*, and *Rhizobium* are established plant growth promoters [18, 24, 25].

This study identified *Acidothermus, Conexibacter, WD2101* soil group, *Mycobacterium, Bryobacter, Sphingomonas, Candidatus_Solibacter, Candidatus_Udaeobacter, TK10*, and *Bacillus* as core genera in both regions, representing 48% of total microbial abundance. Their dominance aligns with adaptability to change, particularly relevant given South Africa's semi-arid climate and the extreme 2015/2016 drought. *Acidothermus* (represented by *A. cellulolyticus*) is prototrophic, thermophilic, cellulolytic, and acidophilic. Its thermotolerance, linked to genes like *HotAldO* [26], is crucial during high temperatures (e.g., up to 33 °C in Mpumalanga, 37 °C in Free State in Dec 2016, exceeding 40 °C off-season).

Though less studied, *Conexibacter* and *Bryobacter* are highly adaptive aerobic chemoorganotrophs found in diverse soils globally, from Russian permafrost [27, 28] to the Sahel [29, 30]. Similarly, *Candidatus_Solibacter* and *Candidatus_Udaeobacter* are globally ubiquitous uncultured genera [31–33]. The mechanisms enabling their global success are largely unknown. However, genomic studies, like that of *Ca. Solibacter usitatus* strain Ellin6076, reveal abundant paralogs involved in metabolism, defence, and regulation [34], suggesting functional diversity aids survival under extreme climatic variation. Furthermore, *Ca. Udaeobacter* scavenges trace gases, exhibits multidrug resistance, and potentially exploits nutrients released by antibiotic-driven lysis of other microbes [33]. If widespread among strains, these strategies could contribute to its global distribution.

*Sphingomonas* (chemoheterotrophic bacteria) are globally ubiquitous, inhabiting diverse environments from contaminated/non-contaminated soil and plant roots to water sources, animals, humans, and even hospital equipment [35–38]. Its remarkable adaptability is evidenced by the prevalence of 13 plant-growth-promoting *Sphingomonas* species across the International Space Station [39]. Pangenome analysis reveals habitat-specific genomic enrichment; strains from plants/environments possess more genes for growth promotion, stress response, and resource acquisition [38]. Survival across habitats is attributed to high stress resistance, unique DNA repair, and minimal nutrient requirements [40]. Other core genera like *Bacillus* and *Mycobacterium* are also highly resilient [41, 42]. They can enter dormancy during nutrient scarcity or unfavourable conditions [41], with *Bacillus* notably forming stress-resilient spores [43]. Drawing from evidence in existing literature, we hypothesize that the prevalence and dominance of specific top genera in both studied regions could be attributed to their superior adaptive characteristics, which enable them to effectively cope with extreme abiotic conditions.

Our study reveals distinct rhizosphere microbial communities in Free State (SAS-FS) and Mpumalanga (SH-M), shaped by contrasting environmental conditions, outweighing uniform farming practices. Significantly higher diversity and richness indices (*P* < 0.05) were observed in SAS-FS compared to SH-M. This divergence is primarily driven by abiotic factors moisture, temperature, and sand content rather than management. Analysis revealed a fundamental ecological divide: non-core ASVs exhibited strong associations with environmental variables, acting as environmentally responsive"opportunists". Conversely, the core community demonstrated remarkable stability across fluctuations, suggesting a conserved, functionally resilient"backbone"potentially maintained by functional redundancy or stress memory mechanisms. This aligns with Mpumalanga's subtropical humidity favouring moisture-sensitive non-core taxa (e.g., Burkholderiaceae, Symbiobacteraceae) for processes like organic matter decomposition, while Free State's semi-arid conditions selected for non-core taxa (e.g., WS2, Legionellaceae) adapted to thermal/osmotic stress and pH/texture via traits like spore formation. Our findings partially align with Kumar et al. [44], who also reported higher richness/diversity in SAS-FS versus SH-M soybean rhizospheres. However, they attributed community differences primarily to pH, nutrient availability, and tillage, contrasting with our observed minimal pH influence on core communities. This divergence highlights critical legacy effects: long-term agricultural practices (tillage, fertilization, monoculture) in managed sites accelerate organic matter mineralization and rhizosphere acidification, amplifying pH's role as a microbial filter. In contrast, the buffering capacity of intact organic matter and microbial networks in our study's maiden soils likely mitigated pH effects. Thus, the impact of specific environmental filters, like pH, is contingent upon land-use history and management intensity.

Soil texture (clay, silt, sand, organic matter) creates distinct microenvironments crucial for specific bacteria, significantly influencing diversity [45]. SAS-FS (Free State) rhizosphere soil had lower sand but higher silt and clay than SH-M (Mpumalanga). Free State's higher clay content retains moisture and nutrients, protecting microbes and favouring slow-growing, stress-tolerant taxa (e.g., Chloroflexi, Verrucomicrobiota) adapted to stable conditions. Conversely, Mpumalanga's sandy soils, with larger pores enhancing oxygen diffusion and lower water retention, promote fast-growing aerobic bacteria (e.g., Burkholderiaceae) in dynamic environments. This aligns with Seaton et al. [46], who found soil textural heterogeneity positively correlates with bacterial diversity, with specific taxa associating with clay/silt particles.

## Conclusion

This study demonstrates that environmental factors exert a direct and significant influence on the structure of soil microbial communities. Specifically, soil texture, moisture, temperature, and pH function as critical selective pressures, differentiating the composition of core and non-core microbial taxa. While non-core amplicon sequence variants exhibited high sensitivity to regional abiotic conditions, the identified core microbial communities comprising dominant phyla such as Actinobacteriota, Pseudomonadota, Chloroflexi, and Acidobacteriota demonstrated remarkable resilience, thereby underscoring their critical role as ecological stabilizers within these agricultural systems. Crucially, our findings emphasize that climate change possesses the potential to indirectly modulate microbial biomass, community composition, and functional diversity through the perturbation of fundamental soil properties, including nutrient retention, organic matter content, and pore structure. Such alterations could consequently precipitate cascading disruptions to essential nutrient cycling processes, bearing profound implications for agroecosystem productivity. Although the core microbial communities exhibit resilience to short-term environmental fluctuations, their long-term stability may be compromised under sustained environmental stress, potentially jeopardizing both soil health and crop resilience. To effectively mitigate these challenges and advance sustainable agricultural practices, future research endeavours should prioritize: (i) Functional characterization of region-specific communities under stressors (e.g., drought, heat) to identify keystone taxa crucial for nutrient cycling and stress tolerance; (ii) Development of climate-adaptive management strategies (e.g., application of drought-resistant Actinobacteriota biofertilizers for clay soils, implementation of precision irrigation for sandy soils); iii) Establishment of long-term monitoring programs for microbial responses to predict ecological tipping points. Ultimately, the strategic integration of microbial ecological principles into agronomic frameworks is paramount for the development of climate-resilient agricultural systems that effectively leverage indigenous microbiomes to enhance productivity and safeguard ecosystem integrity amidst global climate change. Our findings show environmental factors directly.

## Supplementary Information

Below is the link to the electronic supplementary material.

Supplementary file1 (XLSX 107 KB)

Supplementary file2 (XLSX 298 KB)

Supplementary file3 (DOCX 214 KB)

## Data Availability

The obtained Illumina-MiSeq sequence datasets have been deposited in GenBank under the Bio Project number PRJNA667129.An aggregate of weather data used in this study has been provided as supplementary data 1.
