# Supplementary material for: Biogeographical Distribution of Bacteria in Soils with Identical Agricultural Practices: Impacts of Environmental Factors
Source: Curr Microbiol. 2025 Aug 3;82(9):430. doi: 10.1007/s00284-025-04404-w (PMC12318886; doi:10.1007/s00284-025-04404-w)
Supplement: Supplementary file 3 — Supplementary file3 (DOCX 214 KB) [file 284_2025_4404_MOESM3_ESM.docx]

**Biogeographical distribution of bacteria in soils with identical agricultural practices: Impacts of environmental factors**

Muiz O. Akinyemi^1,2^, Sinawo Tsipinana^1^, Kazeem A. Alayande^1^, Maphala Mokubedi^1^, Rasheed A. Adeleke^1,^ *

*^1^Unit for Environmental Sciences and Management, North-West University, Potchefstroom, 2520, South Africa.*

*^2^Leeds Institute of Health Sciences, University of Leeds, Leeds, UK*

*Corresponding author: [Rasheed.Adeleke@nwu.ac.za](mailto:Rasheed.Adeleke@nwu.ac.za)

Journal: Current Microbiology journal


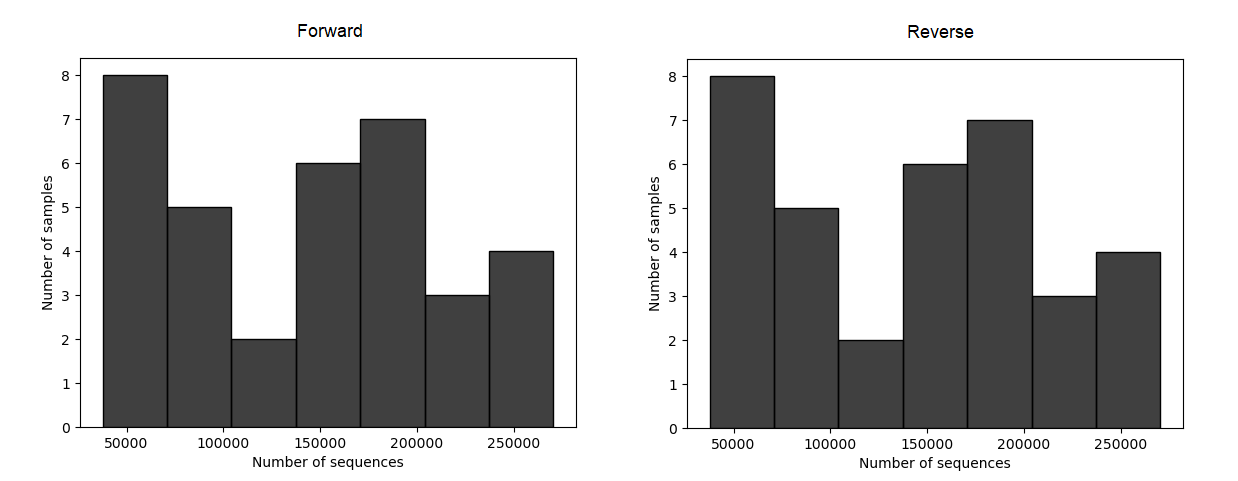
**Supplementary Figure S1:** Barplot indicating the number of sequences recovered from soil samples collected in Free state and Mpumalanga province, South Africa

**
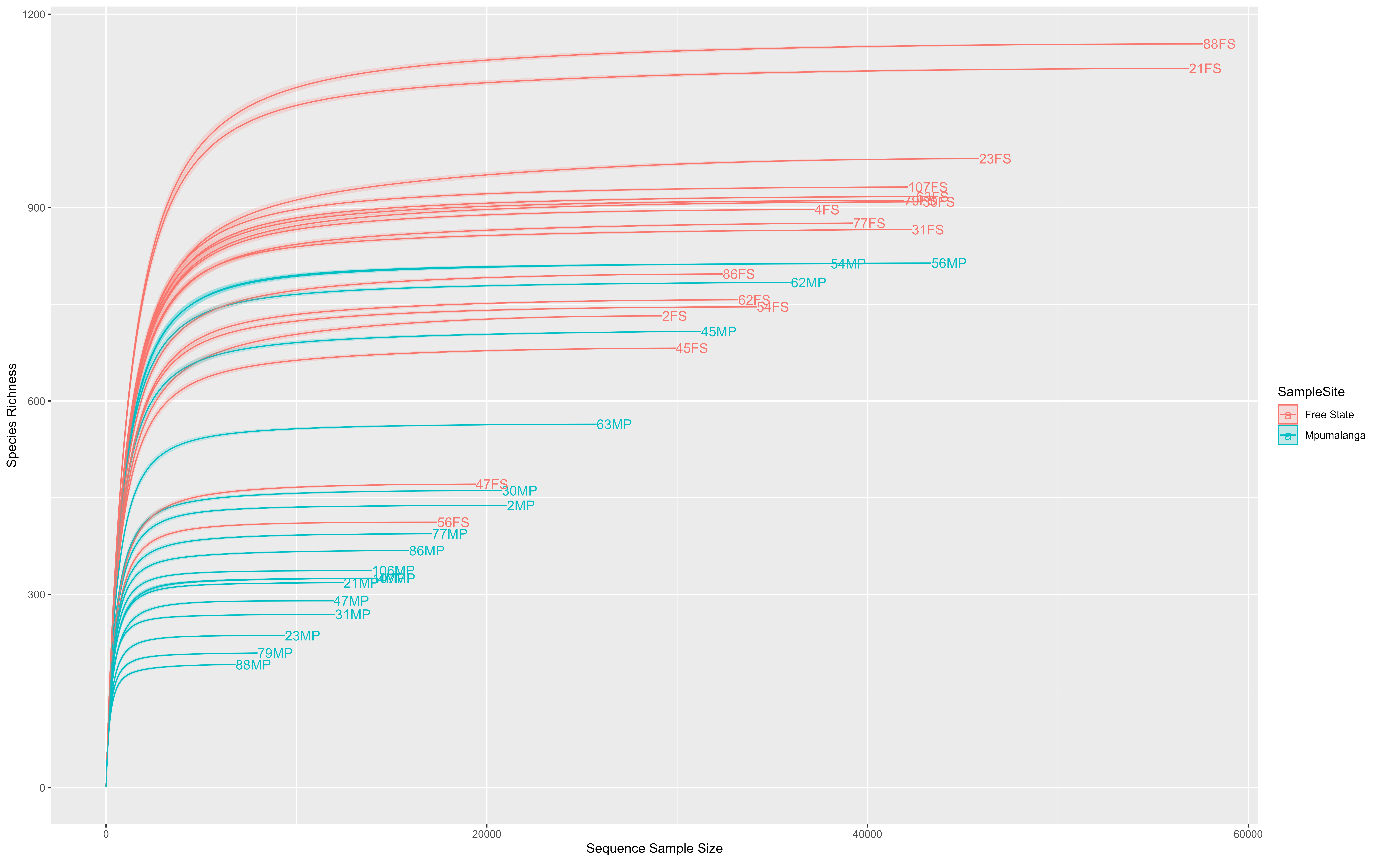
**

Supplementary Figure S2: Rarefaction curves of 16S rRNA genes recovered from soil samples collected in Free state and Mpumalanga province, South Africa


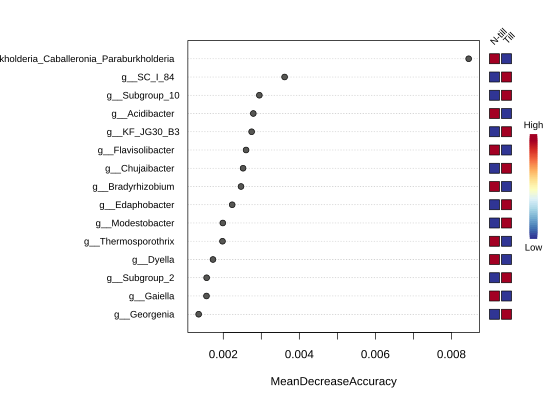


Supplementary Figure S3: Random Forest importance plot of the top 15 genus with the highest importance in till and non-till soil from Mpumalanga province. Features with at least 10 reads and with a minimum prevalence of 10% across samples were included. Data was further transformed to centered log ratio (CLR) before applying the Random Forest classification algorithm
